# Time-Lag in Feeding Schedule Acts as a Stressor That Alters Circadian Oscillators in Goldfish

**DOI:** 10.3389/fphys.2018.01749

**Published:** 2018-12-05

**Authors:** Miguel Gómez-Boronat, Nuria Sáiz, María J. Delgado, Nuria de Pedro, Esther Isorna

**Affiliations:** Departamento de Genética, Fisiología y Microbiología, Unidad Docente de Fisiología Animal, Facultad de Ciencias Biológicas, Universidad Complutense de Madrid, Madrid, Spain

**Keywords:** goldfish, hypothalamus, interrenal tissue, liver, circadian system, food intake, clock genes

## Abstract

The circadian system controls temporal homeostasis in all vertebrates. The light-dark (LD) cycle is the most important *zeitgeber* (“time giver”) of circadian system, but feeding time also acts as a potent synchronizer in the functional organization of the teleost circadian system. In mammals is well known that food intake during the rest phase promotes circadian desynchrony which has been associated with metabolic diseases. However, the impact of a misalignment of LD and feeding cycles in the entrainment of fish circadian oscillators is largely unknown. The objective of this work was to investigate how a time-lag feeding alters temporal homeostasis and if this could be considered a stressor. To this aim, goldfish maintained under a 12 h light-12 h darkness were fed at mid-photophase (SF6) or mid-scotophase (SF18). Daily rhythms of locomotor activity, clock genes expression in hypothalamus, liver, and head kidney, and circulating cortisol were studied. Results showed that SF6 fish showed daily rhythms of *bmal1a* and *clock1a* in all studied tissues, being in antiphase with rhythms of *per1* genes, as expected for proper functioning clocks. The 12 h shift in scheduled feeding induced a short phase advance (4–5-h) of the clock genes daily rhythms in the hypothalamus, while in the liver the shift for clock genes expression rhythms was the same that the feeding time shift (∼12 h). In head kidney, acrophases of *per* genes underwent a 12-h shift in SF18 animals, but only 6 h shift for *clock1a*. Plasma cortisol levels showed a significant daily rhythm in animals fed at SF6, but not in SF18 fish fed, which displayed higher cortisol values throughout the 24-h. Altogether, results indicate that hypothalamus, liver, and head kidney oscillate in phase in SF6 fish, but these clocks are desynchronized in SF18 fish, which could explain cortisol alterations. These data reinforce the hypothesis that the misalignment of external cues (daily photocycle and feeding time) alters fish temporal homeostasis and it might be considered a stressor for the animals.

## Introduction

The circadian system in vertebrates is formed by a widespread network of self-sustainable endogenous clocks located in central and peripheral tissues (Albrecht, 2012; Schibler et al., 2015; Costa et al., 2016; Isorna et al., 2017). These clocks generate circadian endogenous rhythms with a period close, but generally not equal, to 24 h, providing a temporal organization for physiological and behavioral activities making it possible to predict environmental changes (i.e., *zeitgeber*s; Albrecht, 2012; Tsang et al., 2014; Challet, 2015). The most important environmental factor that entrains circadian oscillators is the light-dark (LD) cycle, and clocks synchronized by this *zeitgeber* (“time giver” in German) are named Light-Entrainable Oscillators (LEOs; Reppert and Weaver, 2002; Mendoza and Challet, 2009). However, feeding time is also an important *zeitgeber*, especially for peripheral clocks, and clocks entrained by feeding-fasting cycles are known as Feeding-Entrainable Oscillators (FEOs; Damiola et al., 2000; Mendoza and Challet, 2009).

The circadian clocks machinery is well conserved in vertebrates and it is based on transcriptional-translational feedback loops. The positive limb of the main loop is represented by two transcription factors, CLOCK (Circadian Locomotor Output Cycles Kaput) and BMAL1 (Brain and Muscle ARNT-Like 1), whose heterodimer binds to an E-box rich region in the promoter of the negative limb genes *period* (*per*) and *cryptochrome* (*cry*) (Gekakis et al., 1998; Nakamura et al., 2008). This binding promotes the expression of these last two clock genes, whose products PER and CRY heterodimerize in the cytoplasm and translocate into the nucleus to repress CLOCK-BMAL1 transactivation (Hastings et al., 2007; Nader et al., 2010; Schibler et al., 2015). Moreover, the CLOCK-BMAL1 heterodimer also induces the expression of genes known as clock-controlled genes (CCG), which are considered the outputs of the clock by binding to the E-boxes in their promoters (Hastings et al., 2007; Vatine et al., 2011; Albrecht, 2012). The functioning of this molecular mechanism is conserved, although several copies of these clock genes have been reported in fishes (Vatine et al., 2011; Sánchez-Bretaño et al., 2015a).

In mammals, the master pacemaker is a LEO located in the suprachiasmatic nucleus of the hypothalamus (Reppert and Weaver, 2002; Welsh et al., 2010) that controls in an hierarchical manner the rest of pacemakers widely distributed over the organisms (Dibner et al., 2010). It is evident that the organization of the circadian system in fish is less hierarchical than in mammals, since a master clock has not been clearly identified yet (Moore and Whitmore, 2014; Sánchez-Bretaño et al., 2015a; Isorna et al., 2017). Despite the greater or lesser hierarchical role of central pacemakers, evidences of the physiological relevance of peripheral circadian clocks in vertebrates are emerging. It is suggested that the entrainment of peripheral clocks by feeding-fasting cycles allows peripheral tissues to anticipate food supply, and potentially optimizing processes required for food digestion, metabolism, and energy storage and utilization (Vera et al., 2007; Lamia et al., 2008). Indeed, food intake during the rest phase promotes circadian desynchrony, which has been associated with metabolic diseases in mammals (Ferrell and Chiang, 2015; Ramirez-Plascencia et al., 2017), thus a time-lag feeding schedule can be considered a stressor that alters temporal homeostasis. In fish, feeding time is a potent *zeitgeber* for peripheral oscillators of the gastrointestinal tract (Isorna et al., 2017). In fact, feeding time affects daily locomotor activity rhythms (Aranda et al., 2001; Cavallari et al., 2011; Feliciano et al., 2011); clock genes expression in liver, gut, and encephalic tissues (López-Olmeda et al., 2009, 2010; Feliciano et al., 2011; Nisembaum et al., 2012; Tinoco et al., 2014); and daily profile of circulating cortisol (Montoya et al., 2010; Cowan et al., 2017). But a variety of results are obtained depending on species and protocols employed (Cowan et al., 2017). Nevertheless, the effect of feeding time on the clock of the interrenal tissue has not been investigated in any fish species to date, and it is unknown if this oscillator behaves as a LEO or a FEO. In fact, the paradigm of a time-lag in feeding schedule and its consequences in locomotor activity, peripheral oscillators and cortisol production has not been studied all at once and in the same species.

Therefore, the aim of this work was to study, if a time-lag in scheduled feeding alters temporal homeostasis in fish and to test its possible role as a stressor. To this end, we have studied the effects of 12 h shifted feeding schedule on daily expression of clock genes in the hypothalamus and two peripheral oscillators, the liver and the head kidney in goldfish (*Carassius auratus*). We have also investigated if this paradigm affects circulating cortisol daily rhythms as stress indicator and hepatic leptin expression as a putative output of the liver clock. The interest to study such oscillators is based on several reasons. The hypothalamus plays a key role in the control of both, energy homeostasis and the hypothalamus-pituitary-interrenal (HPI) axis, acting as an integrative core of environmental and endogenous signals. The role of the liver as a nexus between metabolism and circadian system in mammals and fish has been outlined (Albrecht, 2012; Schmutz et al., 2012; Tsang et al., 2014; Schibler et al., 2015), emphasizing this tissue as a key food-sensitive clock. Finally, the interrenal tissue (contained in the head kidney) is the main source of cortisol, which initiates the stress response (Schreck and Tort, 2016), and its daily rhythm is considered as the most robust hormonal rhythmic output in vertebrates (Isorna et al., 2017; Spencer et al., 2018).

## Materials and Methods

### Animals and Housing

Goldfish (*C. auratus*) with a body weight (bw) of 24 ± 5 g were obtained from a local commercial supplier (ICA, Madrid, Spain). Fish were housed in 60 l aquaria with filtered and aerated fresh water (21 ± 2°C) under a 12 h light and 12 h darkness (12L:12D) photoperiod (lights on at 8 am, considered as *Zeitgeber* Time 0, ZT 0). Fish were fed with automatic feeders that daily delivered food pellets (1% bw; Sera Pond Biogranulat, Heinsberg, Germany) at ZT 2. Animals were acclimated during 2 weeks under these conditions before the beginning of the experiments. The experiments comply with the Guidelines of the European Union Council (UE63/2010), and the Spanish Government (RD53/2013) for the use of animals in research and were approved by the Animal Experimentation Committee of Complutense University (O.H.-UCM-25-2014), and the Community of Madrid (PROEX 107/14).

### Experimental Design

Two groups of fish maintained under the same 12L:12D photoperiod (lights on at 8 a.m.) were fed with different schedules with automatic feeders to avoid the negative effects of the human feeding activities. One group (*n* = 36, placed in six aquaria, six fish/tank) was daily fed at mid-photophase (ZT 6, named Scheduled Feeding 6, SF6), and the other one (*n* = 36, placed in six aquaria) was daily fed at mid-scotophase (ZT 18, named SF18). Three weeks later, goldfish were sampled each 4 h throughout a 24 h cycle (one tank (*n* = 6) per sampling time at ZT 5, ZT 9, ZT 13, ZT 17, ZT 21, and ZT 1). Blood was collected from the caudal vein of anesthetized animals (tricaine methanesulfonate, MS-222, 0.14 g/l; Sigma-Aldrich, Madrid, Spain), and plasma was obtained after blood centrifugation and stored at -80°C until assay. Fish were then sacrificed by anesthetic overdose (MS-222, 0.28 g/l), and hypothalamus, head kidney, and liver were quickly collected, frozen in liquid nitrogen and stored at -80°C until analysis.

### Locomotor Activity Recordings

Daily locomotor activity was recorded during the experimental period by six infrared photocells (Omron Corporation, E3S-AD12, Japan) fixed on the walls of each aquarium wall. Two photocells were located below the automatic feeder (for recording feeding-related activity), while the remaining four photocells were placed at a height of 3–9 cm above the bottom in each aquaria wall (for recording general locomotor activity). With this arrangement of photocells, we obtained reproducible actograms, more photocells increase the total amount of activity but does not affect daily profiles. Each photocell continuously emitted an infrared light beam which was interrupted each time fish swam in that zone, generating an output signal. The number of light beam interruptions was automatically registered every 10 min by a computer with specific software (Micronec, Spain). The aquaria walls were covered with opaque paper to minimize external interferences during the experiment. Data were analyzed using the chronobiology software EL TEMPS^®^(Prof. Antoni Díez Noguera, University of Barcelona), and actograms and periodograms were performed.

### Gene Expression Analysis

Total RNA from hypothalamus, head kidney, and liver were isolated using TRI^®^Reagent (Sigma-Aldrich) and treated with RQ1 RNase-Free DNase (Promega, Madison, United States) according to the manufacturer’s instructions. Then, 0.3 μg of total RNA was reverse transcribed into cDNA in a 25 μl reaction volume using random primers (Invitrogen, Carlsbad, United States), RNase inhibitor (Promega), and SuperScript II Reverse Transcriptase (Invitrogen). The reverse transcription reaction conditions consisted of an initial step at 25°C for 10 min, an extension at 42°C for 50 min, and a denaturalization step at 70°C for 15 min. Real-Time quantitative PCRs (RT-qPCRs) were carried out by duplicate in a CFX96 Real^TM^-Time System (Bio-Rad Laboratories, Hercules, United States), using iTaq^TM^ Universal SYBR^®^Green Supermix (Bio-Rad Laboratories) using a 96-well plate loaded with 1 μl of cDNA and a final concentration of 0.5 μM of each forward and reverse primers in a final volume of 10 μl. Each PCR run included also a four-points serial standard curve, non-retrotranscribed-RNA (as positive control) and water (as negative control). The RT-qPCR cycling conditions consisted of an initial denaturation at 95°C for 30 s and 40 cycles of a two-step amplification program (95°C for 5 s and 60°C for 30 s). A melting curve was systematically monitored (temperature gradient at 0.5 C/5 s from 70 to 90°C) at the end of each run to confirm the specificity of the amplification reaction. The Gene Data Bank reference numbers and the primers (Sigma-Aldrich) sequences employed for target genes (clock genes: *per1a, per1b, per2a, per3, bmal1a*, and *clock1a*; and *leptin aI)* and the reference gene (*ef*-*1α*) are shown in Table 1. The 2^-ΔΔ^*^C^*^t^ method (Livak and Schmittgen, 2001) was used to determine the relative mRNA expression (fold change). Data obtained were normalized to the group with the lowest expression in each gene.

**Table 1:** Accession numbers of the genes and primers sequences employed in quantitative RT-qPCR studies.

Gene	Accession number		Primer sequence 5′→3′	Product (bp)
*per1a*	EF690698	Forward	CAGTGGCTCGA ATGAGCACCA	155
		Reverse	TGAAGACCTG CTGTCCGTTGG	
*per1b*	KP663726	Forward	CTCGCAGCTC CACAAACCTA	235
		Reverse	TGATCGTGCA GAAGGAGCCG	
*per2a*	EF690697	Forward	TTTGTCAATC CCTGGAGCCGC	116
		Reverse	AAGGATTTGC CCTCAGCCACG	
*per3*	EF690699	Forward	GGCTATGGCAGT CTGGCTAGTAA	130
		Reverse	CAGCACAAAAC CGCTGCAATGTC	
*bmal1a*	KF840401	Forward	AGATTCTGTT CGTCTCGGAG	161
		Reverse	ATCGATGAGTC GTTCCCGTG	
*clock1a*	KJ574204	Forward	CGATGGCAGC ATCTCTTGTGT	187
		Reverse	TCCTGGATCTG CCGCAGTTCAT	
*leptin aI*	FJ534535	Forward	AGCTCCTCA TAGGGGATC	192
		Reverse	TAGATGTCGTT CTTTCCTTA	
*ef-1α*	AB056104	Forward	CCCTGGCCA CAGAGATTTCA	101
		Reverse	CAGCCTCGAA CTCACCAACA	


*per* period; *bmal1a*, brain and muscle ARNT-like 1a; *clock1a* circadian locomotor output cycles kaput 1a; *ef-1α*, elongation factor-1α.

### Plasma Cortisol Assay

Plasma cortisol levels were determined by enzyme–linked immunosorbent assay (ELISA) using a commercial kit (Demeditec, Schleswig-Holstein, Germany), previously validated for goldfish plasma (Azpeleta et al., 2010). The lowest analytical detectable level of cortisol that can be distinguished from the zero calibrator was 3.79 ng/ml. Free cortisol values were expected to be within the range described by the manufacturer (10–800 ng/ml), therefore no dilution was necessary.

### Data Analysis

The existence of significant periods in daily locomotor activity was analyzed by constructing chi-square periodograms with a significance level set at 0.05 (EL TEMPS^®^). A one-way ANOVA followed by the *post hoc* Student-Newman-Keuls (SNK) test was performed to compare data obtained for gene expression and cortisol levels at different sampling points (using SigmaPlot 12.0 statistics package). When necessary, data were transformed to logarithmic or square root scale to normalize and to obtain homoscedasticity. Statistical differences among groups were noted with different letters. In addition, we have performed a Mann-Whitney *U* Test for analyzing the differences between the mean of cortisol levels in fish fed at ZT 6 and ZT 18. A probability level of *p* < 0.05 was considered statistically significant in all tests. Daily (24 h) significant rhythms in gene expression and cortisol were determined by Cosinor analysis fitting the data to sinusoidal functions by the least squares method (Duggleby, 1981). The formula used was *f(t)* = M+Acos(tπ/12-Φ), where *f(t)* is the gene expression level at a given time, the mesor (M) is the mean value, A is the sinusoidal amplitude of oscillation, t is time in hours, and Φ is the acrophase (time of peak expression). Non-linear regression allows the estimation of M, A, Φ, and their standard errors (SE), which are calculated on the residual sum of squares in the least-squares fit (Duggleby, 1981; Delgado et al., 1993). Significance of Cosinor analysis was defined by the noise/signal of amplitude calculated from the ratio *SE(A)/A* (Nisembaum et al., 2012).

## Results

### Effects of Feeding Time on Synchronization of Locomotor Activity Daily Rhythms

**FIGURE 1:**
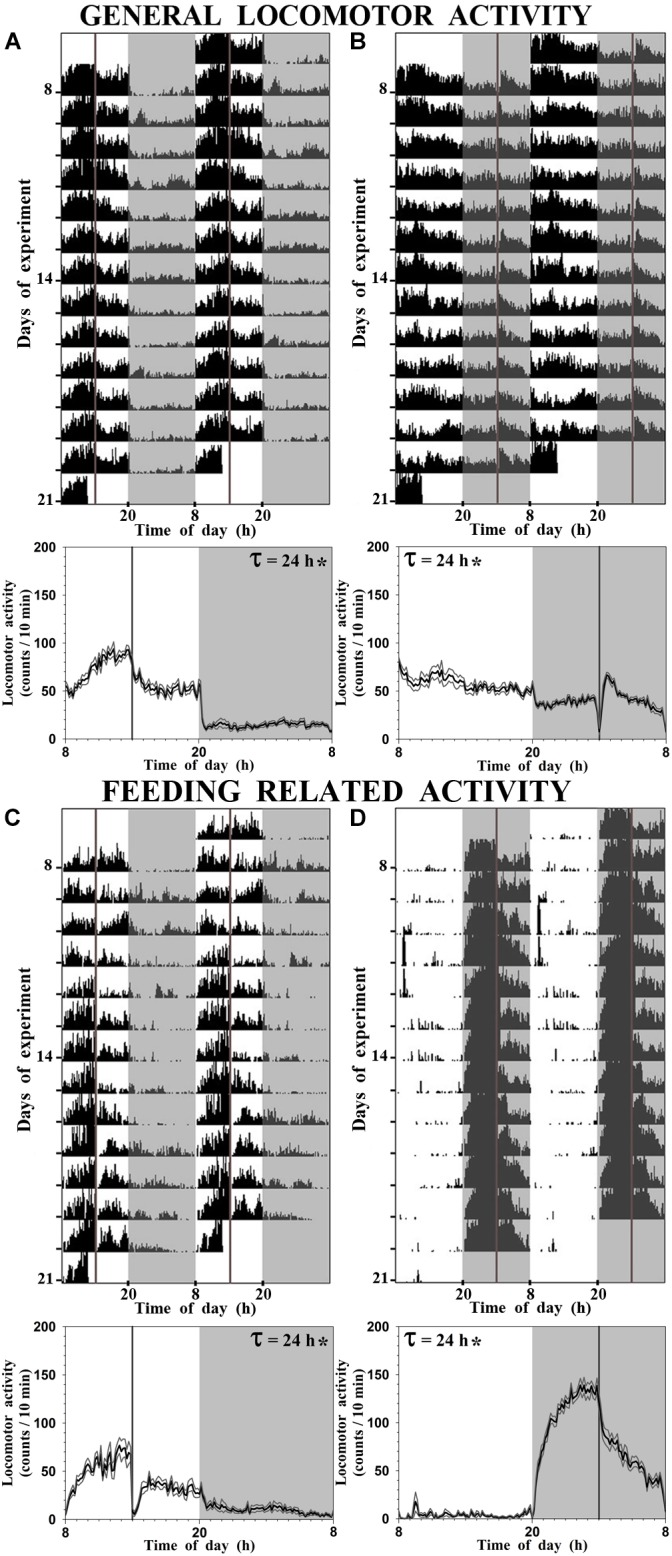
Representative actograms and daily average waveform of goldfish maintained under a 12L:12D photoperiod and fed at ZT 6 [SF6, **(A**,**C)**] or at ZT 18 [SF18, **(B,D)**]. For convenient visualization, data in the actograms have been double-plotted in a time scale of 48 h. **(A,B)**, general locomotor activity; **(C,D)**, feeding-related locomotor activity. In the average waveform graphs, black line represents the mean, gray lines represent the SEM, and τ indicates the period of the rhythm when significant (^∗^).

Daily locomotor activity was registered during 14 days before sampling. Representative double-plotted actograms with the general locomotor activity of fed fish at ZT 6 and ZT 18 are shown in Figures 1A,B, respectively, while the feeding-related activity is shown in Figure 1C (SF6) and Figure 1D (SF18). General activity of SF6 goldfish displayed a diurnal significant rhythm (evidenced by a significant 24 h period; Figure 1A), with higher general activity during the photophase (80% of total activity). As expected, the feeding-related activity was concentrated around 3–4 h before scheduled feeding, corresponding to the food anticipatory activity (FAA), with a significant daily rhythm with a period of 24 h (Figure 1C). When scheduled feeding time was shifted to the mid-scotophase, the general locomotor activity remained rhythmic (period of 24 h), but its 24 h profile was flattened (Figure 1B), and surprisingly general locomotor activity continued being higher during the photophase (60% of total activity). Nevertheless, fish fed at ZT 18 showed a robust FAA during the night with a significant daily rhythm (period of 24 h; Figure 1D).

### Daily Rhythms of Clock Genes Expression in Goldfish

**FIGURE 2:**
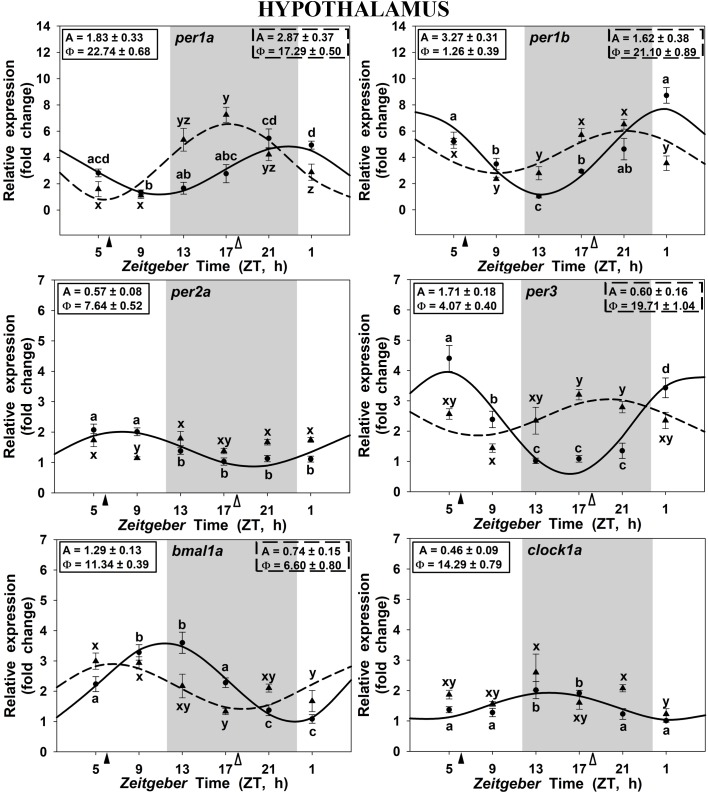
Daily profile of clock genes expression in the hypothalamus of SF6 (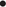) and SF18 (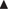) goldfish maintained under a 12L:12D photoperiod. Gray area indicates the dark period while feeding time is indicated by triangles in the x-axis (solid, ZT 6; white, ZT 18). Data obtained by RT-qPCR are shown as mean ± SEM (*n* = 6) in relative units (2^-ΔΔ^*^C^*^t^ method). Different letters (**a–d** in SF6 and **x–z** in SF18) indicate significant differences. When Cosinor [*SE(A)/A* < 0.3] was significant, periodic sinusoidal functions were represented as solid waves (SF6 fish) or dashed waves (SF18 fish), and amplitudes and acrophases (A and Φ, respectively) are shown at the top of the panels (SF6, left; SF18 right).

In the hypothalamus of SF6 animals, all studied genes exhibited significant 24 h rhythms (Figure 2), with acrophases of *per1* genes at the end of the dark phase (ZT 22.7 for *per1a*; Figure 2A) and at the light onset (ZT 1.2 for *per1b*; Figure 2B). These rhythmic profiles are in antiphase with those shown by *bmal1a* (ZT 11.3; Figure 2E) and *clock1a* (ZT 14.3; Figure 2F). Hypothalamic *per3* expression in the SF6 fish peaked around ZT 4 (Figure 2D), while the maximum expression of *per2a* occurred at midday (ZT 7.6; Figure 2C). The expression profiles of the clock genes in the scheduled-fed goldfish at ZT 18 also showed 24 h rhythms in the hypothalamus (Figures 2A,B,D,E), except for *per2a* and *clock1a*, whose rhythms were lost (Figures 2C–F). The shift in the scheduled feeding time from ZT6 to ZT18 advanced 4–5 h the acrophases in the case of *per1a, per1b*, and *bmal1a* genes, and 9 h for *per3* (Figures 5A,B) in hypothalamus.

**FIGURE 3:**
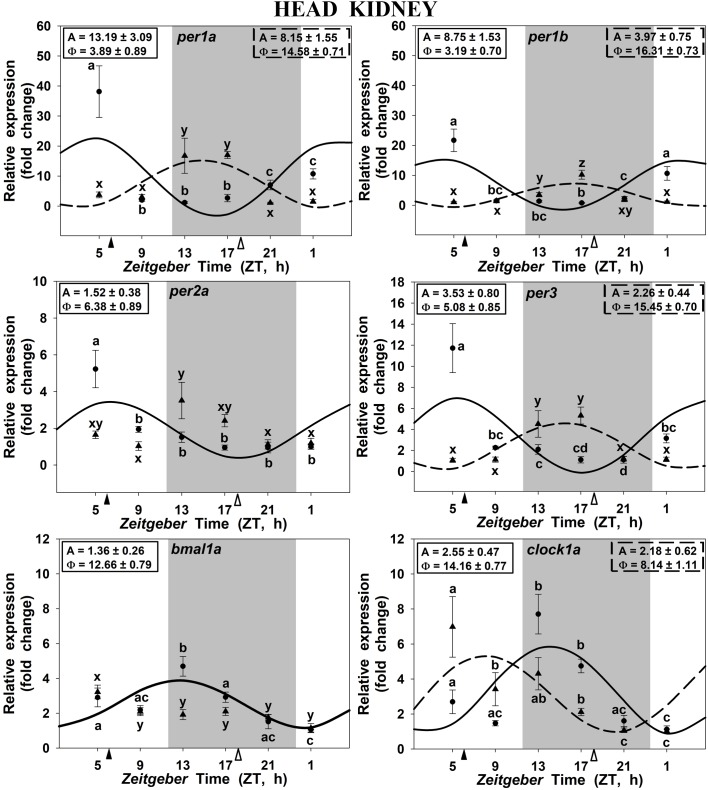
Daily profile of clock genes expression in the head kidney of SF6 (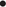) and SF18 (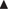) goldfish maintained under a 12L:12D photoperiod. Gray area indicates the dark period while feeding time is indicated by triangles in the x-axis (solid, ZT 6; white, ZT 18). Data obtained by RT-qPCR are shown as mean ± SEM (*n* = 6) in relative units (2^-ΔΔ^*^C^*^t^ method). Different letters (**a–c** in SF6 and **x–z** in SF18) indicate significant differences. When Cosinor [*SE(A)/A* < 0.3] was significant, periodic sinusoidal functions were represented as solid waves (SF6 fish) or dashed waves (SF18 fish), and amplitudes and acrophases (A and Φ, respectively) are shown at the top of the panels (SF6, left; SF18 right).

In the head kidney, all examined clock genes showed significant daily variation in their expression in both groups of scheduled-fed goldfish (SF6 and SF18; Figure 3), with the exception of *per2a* and *bmal1a*, which lost their significant daily rhythmicity when scheduled feeding was shifted from midday to midnight (Figures 3C–E). The daily expression profiles in the head kidney of SF6 fish were broadly similar to the rhythms observed in the hypothalamus, with similar acrophases, as it can be observed in polar graphs (Figures 5A–D). However, a slight shift seems to exist for *per1b* and *per1a* in the head kidney of SF6 fishes compared to the hypothalamus of the same animals (Figures 5A–C). The amount of *per1* transcripts peaked at the early morning, which is in antiphase with the expression of *bmal1a* and *clock1a*, whose acrophases were located at the end of the light phase and beginning of the dark phase, as occurs in the hypothalamus. Thus, hypothalamic and head kidney oscillators seem to be in phase in SF6 fish. In contrast to the minor effect observed in the hypothalamus, the 12 h-shift in feeding schedule produced a complete shift (11–13 h) in *per1* and *per3* rhythms in the head kidney of goldfish, but only a 6 h advance for *clock1a*, suggesting that these negative and positive elements of the head kidney clock were not in antiphase. The expression of *per2a* showed a significant rhythm in the head kidney of SF6 but not in SF18 fish, as occurs in the hypothalamus, with similar acrophases in both tissues.

**FIGURE 4:**
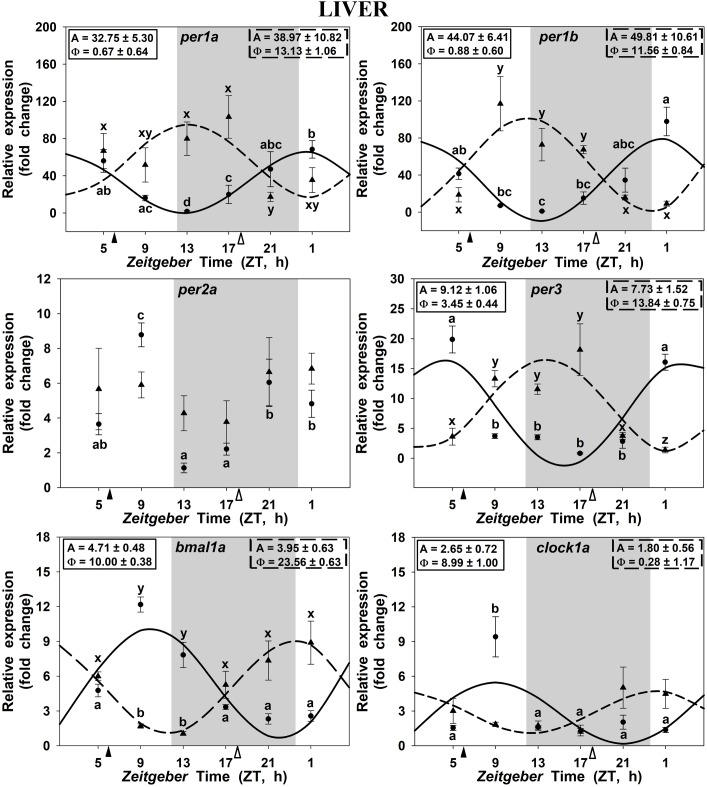
Daily profile of clock genes expression in the liver of SF6 (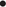) and SF18 (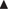) goldfish maintained under a 12L:12D photoperiod. Gray area indicates the dark period while feeding time is indicated by triangles in the x-axis (solid, ZT 6; white, ZT 18). Data obtained by RT-qPCR are shown as mean ± SEM (*n* = 6) in relative units (2^-ΔΔ^*^C^*^t^ method). Different letters (**a–c** in SF6 and **x–z** in SF18) indicate significant differences. When Cosinor [*SE(A)/A* < 0.3] was significant, periodic sinusoidal functions were represented as solid waves (SF6 fish) or dashed waves (SF18 fish), and amplitudes and acrophases (A and Φ, respectively) are shown at the top of the panels (SF6, left; SF18 right).

**FIGURE 5:**
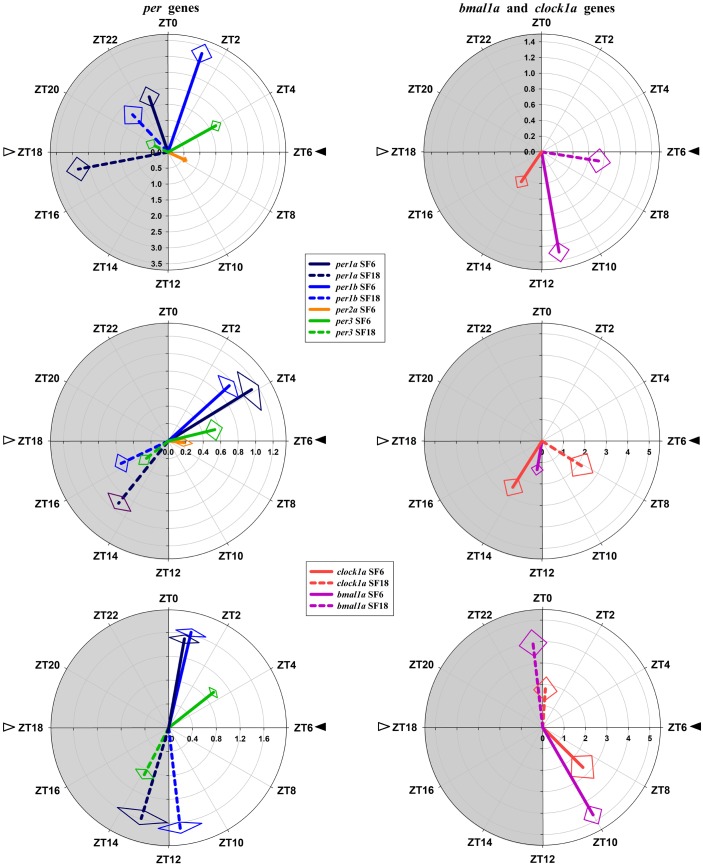
Polar representations of parameters defining clock genes rhythms. **(A,B)** hypothalamus, **(C,D)** head kidney, **(E,F)** liver. The length of the vector (radial axis) indicates the value of the amplitude [fold change of relative expression, **C,E** in logarithmic scale]. The angular position indicates the acrophase (ZT, zeitgeber time). The SE of these two parameters is represented by the rhombus at the end of each vector.

Clock genes expression in the goldfish liver displayed significant 24 h rhythms in both SF6 and SF18 fish (Figure 4), except for *per2a*, which did not show daily rhythmicity in any studied groups (Figure 4C). In SF6 animals, rhythmic profiles of clock genes expression were similar to those observed in the hypothalamus and the head kidney. The acrophases of *per1* rhythms are located at the light onset (ZT 0.7 and ZT 0.9 for *per1a* and *per1b*, respectively; Figures 4A,B) or the early morning (ZT 3.4 h for *per3*; Figure 4D), which is in antiphase with *bmal1a* (ZT 10.0) and *clock1a* genes (ZT 9.0; Figures 4E,F, 5E,F). When feeding schedule was shifted from midday to midnight, all clock genes also underwent a 12 h shift in their acrophases, being moved to the LD transition in the case of *per* genes and to the light onset for *bmal1a* and *clock1a* genes (Figures 4, 5). Thus, the hepatic oscillator seems to be in phase (i.e., positive elements *vs.* negative elements) in both SF6 and SF18 fishes, as in the hypothalamus, but not in the head kidney.

Comparing the clocks in the three analyzed tissues, in SF6 animals these clocks ticked at time (i.e., clock genes are in phase in the different tissues). However, acrophases of clock genes rhythms in the hypothalamus of SF18 animals were in antiphase with the hepatic ones, being the head kidney oscillator in an intermediate condition. Another different aspect of the liver oscillator, compared to the hypothalamus, and the head kidney, is referred to the amplitudes of the genes, which were much higher in the liver. In this sense, the amplitudes of *per* genes were more than 10 times higher than in the hypothalamus and about 3–5 times higher than in the head kidney in both SF6 and SF18 animals.

### Daily Rhythms of Circulating Cortisol and Leptin Expression in the Liver

**FIGURE 6:**
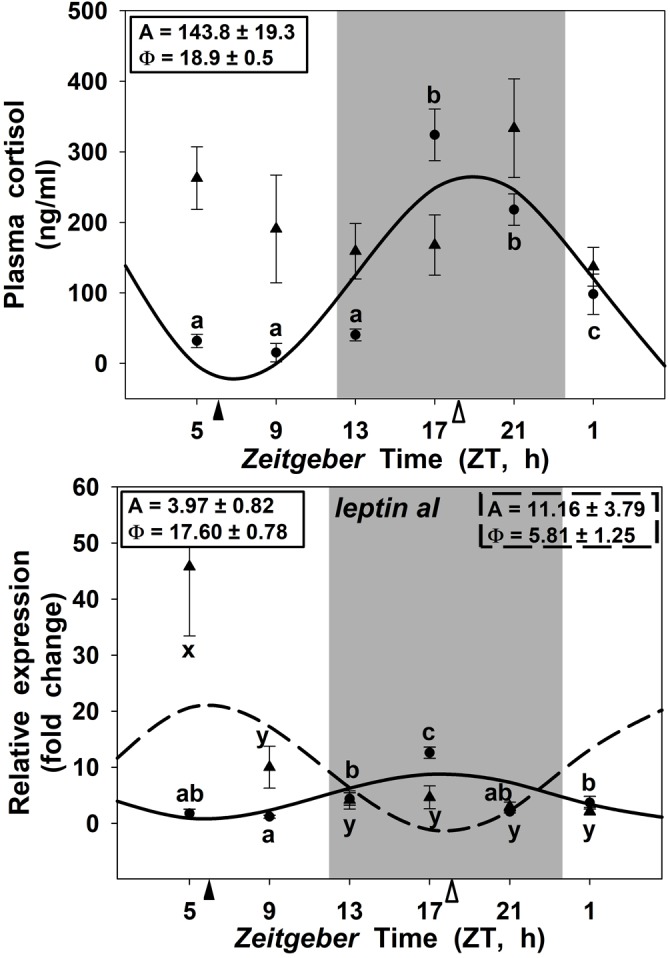
Daily profiles of plasma cortisol **(A)** and *leptin-aI* expression in the liver **(B)** of SF6 (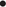) and SF18 (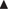) goldfish maintained under a 12L:12D photoperiod. Gray area indicates dark period and feeding time is indicated by triangles in the x-axis (solid, ZT 6; white, ZT 18). Data are shown as mean ± SEM (*n* = 6). Different letters (**a–c** in SF6, and **x–z** in SF18) indicate significant differences. When Cosinor [*SE(A)/A* < 0.3] was significant, periodic sinusoidal functions were represented as solid waves (SF6 fish) or dashed waves (SF18 fish), and amplitudes and acrophases (A and Φ, respectively) are shown at the top of the panels (SF6, left; SF18 right).

Circulating cortisol displayed a significant daily rhythm in goldfish fed at midday with a robust amplitude (143.8 ng/ml) and the acrophase during the scotophase (at ZT 18.9; Figure 6A) 6 h before lights on. By contrast, in the SF18 group this 24 h rhythmicity was fully abolished. Moreover, the SF18 fed fish showed significantly higher levels of cortisol (202.19 ± 22.78 ng/ml) than that observed in SF6 fed fish (126.95 ± 23.06 ng/ml) (*p* < 0.05, Mann-Whitney *U* Test). Hepatic *leptin aI* expression showed significant daily rhythms in both SF6 and SF18 fish (Figure 6B). The acrophase of *leptin aI* rhythm was found at the middle of the scotophase (ZT 17.6) in fish fed at ZT 6, while it was shifted at midday (ZT 5.8) in SF18 fish. Thus, the 12-h-shift in feeding schedule from midday to midnight induced a 12-h shift in the rhythmic expression of *leptin aI* in goldfish liver.

## Discussion

Results obtained clearly show that a shift in feeding schedule alters temporal homeostasis in goldfish, as it differently affects clocks (i.e., clock genes expression rhythms) in the hypothalamus, the liver, and the head kidney. In fish fed at midday, these three oscillators tick at time with similar acrophases for each gene in the different tissues. However, in fish fed at mid-scotophase, daily expression rhythms of clock genes are not in phase in the different tissues, and *per1* and *clock-bmal* genes do not follow their characteristic profiles of expression in antiphase, particularly in the head kidney. Then, time-lag in feeding schedule seems to represent a stressor for the animals, since alters the temporal homeostasis, with increases in plasma cortisol and the disappearance of its daily rhythm in fish fed in the mid-scotophase.

It is widely known that food acts as a potent *zeitgeber* for circadian rhythms when restricted or provided on a periodic basis (Hara et al., 2001; Stephan, 2002). As expected, goldfish adapted their daily locomotor activity to feeding schedule; SF6 fish showed a robust FAA in the photophase while SF18 fish showed it during the scotophase. It is previously reported that a scheduled feeding under continuous light (Vera et al., 2007; Feliciano et al., 2011), at the start or the end of the photophase (Aranda et al., 2001), or at the beginning of the scotophase (Vivas et al., 2011) synchronizes daily activity to feeding time in goldfish. However, it has been also reported that if both *zeitgeber*s are present, both are important (Aranda et al., 2001). In this sense, our data revealed that SF6 goldfish are clearly diurnal (80% of the activity during the photophase), but SF18 fish has not became nocturnal, since they reduce their locomotor activity during daytime but remain active through the 24 h. In fact, they continue to move more during the photophase (60%) than during the scotophase. Thus, it seems that goldfish is not as flexible as previously suggested in terms of daily activity pattern (Isorna et al., 2017). Currently, it is not possible to discern if the alteration of locomotor activity rhythm in SF18 goldfish is related to the time-lag observed in clock genes expression, or if it is due to the loss of cortisol rhythm. Further studies are needed to assess such possibilities.

In fish fed at midday (ZT 6), the *per1a* and *per1b* genes in the hypothalamus, the head kidney and the liver displayed significant daily rhythms with their acrophases at the onset of the photophase or at the end of the scotophase, in accordance with previous reports in goldfish also maintained in 12L:12D and fed during the photophase at ZT 2 (Velarde et al., 2009; Nisembaum et al., 2012; Sánchez-Bretaño et al., 2015b). Similarly, a *per1* peak around the dark-light transition has been also reported in other teleosts, as zebrafish brain (*Danio rerio*; Sanchez and Sanchez-Vazquez, 2009; Vatine et al., 2011), European sea bass brain and liver (*Dicentrarchus labrax*; Sánchez et al., 2010), rainbow trout hypothalamus (*Oncorhynchus mykiss*; Patiño et al., 2011), Senegalese sole retina and optic tectum (*Solea senegalensis*; Martín-Robles et al., 2012), or Nile tilapia brain (*Oreochromis niloticus*; Costa et al., 2016). All these findings support the hypothesis that *per1* genes anticipate the light arrival in fish under these conditions (Isorna et al., 2017). Moreover, the clock genes of the positive limb of the loop (*bmal1a* and *clock1a*) were in antiphase with the negative limb genes (*per*) in these three tissues, showing their acrophases almost in the LD interphase, as previously reported in goldfish (Nisembaum et al., 2012), and other fish species under a LD photocycle (Patiño et al., 2011; Vatine et al., 2011; Martín-Robles et al., 2012; Costa et al., 2016).

Is feeding time able to modify such clock genes rhythmicity? As previously mentioned, food acts as a potent *zeitgeber* nor only for circadian activity rhythms (Aranda et al., 2001; Stephan, 2002; López-Olmeda et al., 2009; Refinetti, 2015) but also for clock synchronization (Damiola et al., 2000; Feliciano et al., 2011; Nisembaum et al., 2012) in mammals and fish. Our findings revealed that feeding time exerts different effects on clock genes expression at central and peripheral levels. In the hypothalamus, a 12 h shift in the feeding schedule (adjusting the feeding time at the mid-scotophase) induced a minor shifting of 4–5 h in the acrophases of the target genes (except *per2a* as expected and below discussed), in agreement with previous reports in the European sea bream (*Sparus aurata*; (Vera et al., 2013), and the Nile tilapia brain (Costa et al., 2016). These findings indicate that feeding time is able to induce a slight displacement of the acrophases, but the LD cycle seems to be the main synchronizer of the rhythmic expression of hypothalamic clock genes, as previously suggested (Hara et al., 2001; Sanchez and Sanchez-Vazquez, 2009; Feliciano et al., 2011; Nisembaum et al., 2012; Tinoco et al., 2014). Interestingly, the amplitudes of the central clock genes were diminished when the food was supplied at midnight (except for *per1a*), suggesting that feeding-fasting cycles enhance LD driven-daily rhythms, in agreement with previous reports (Sánchez-Bretaño et al., 2015a).

It is worthy to highlight the case of *per2a*, the only gene that did not change its expression pattern in any of the three studied tissues when feeding time was shifted. Previous reports have shown that *per2a* displayed a rhythmic expression in some central and peripheral tissues of goldfish, under a LD cycle with acrophases at midday (Velarde et al., 2009; Nisembaum et al., 2012), as in sea bass brain (Herrero and Lepesant, 2014). Such rhythms usually disappear in constant conditions, light or darkness (Feliciano et al., 2011; Nisembaum et al., 2012; Vera et al., 2013), showing that *per2a* rhythmicity is strongly dependent of the LD cycle. Indeed, it is well-known that *per2a* is a light-induced gene with a key role in the molecular mechanism that entrains the LEOs in zebrafish (Vatine et al., 2011; Moore and Whitmore, 2014; Ben-Moshe et al., 2014; Ceinos et al., 2018). Our results support this role of *per2a* as a light-dependent clock gene also in goldfish.

A substantial finding is the 12 h shifting in the acrophases of all hepatic clock genes when feeding time was shifted 12 h (from midday to midnight). Unlike in the hypothalamus, amplitudes of all rhythms shown by the different clock genes in the liver were not significantly affected by feeding time. Vera et al. (2013) obtained comparable results, reporting a 6–7 h shifting in the liver of sea bream fed at mid-photophase compared to fish fed at the mid-scotophase. All these data point out that feeding time is a synchronizer powerful than the LD cycle in the liver, as it is previously proposed in mammals (Damiola et al., 2000; Stokkan et al., 2001; Kornmann et al., 2007). This conclusion was also suggested by Feliciano et al. (2011), who demonstrate significant rhythms for clock gene expression driven by the last meal, independently of previous feeding approaches (random or scheduled feeding). Therefore, the hepatic clock might be a peripheral FEO in goldfish. In terms of adaptation to the new scheduled feeding, the shift in clock genes expression could be an advantage for the animal physiology. However, overt rhythms (i.e., outputs of the circadian system) are complex and usually dependent of more than one oscillator. Thus, although liver clock genes are synchronized to receive food at mid-scotophase, metabolic rhythms could not be adapted. In this sense, lipid metabolism rhythmicity is linked to the LD cycle, independently of feeding time in zebrafish and sea bream liver (Paredes et al., 2014, 2015), although feeding time drives clock genes oscillations in the last species (Vera et al., 2013). Surprisingly, our results show that hepatic leptin expression rhythms match with clock genes expression rhythms in liver, and the acrophase is 12 h shifted in SF6 compared to SF18 animals. This suggests that maybe not all of the metabolic outputs are driven by the same *zeitgebers* in the liver of goldfish.

Regarding the head kidney, fish fed at midday exhibit significant daily rhythms in the expression of all clock genes, with genes of the positive and negative limbs of the loop in antiphase (except *per2a*, as above discussed), confirming the existence of a functional clock in this tissue, as in the adrenal gland of mammals (Son et al., 2008; Kwon et al., 2011). Even though, the interrenal tissue of goldfish is not directly related to the gastrointestinal system, feeding time seems to play an important role on its synchronization, since the expression of *per1* genes had a peak just before the expected feeding time in both experimental groups (at ZT∼4 when food was provided at ZT 6, and at ZT∼15 when provided at ZT 18). Hence, the 12 h time-lag in the feeding time shifted the rhythmic expression pattern of *per1* genes, similarly as the liver’s response. This is not surprising, given that several peripheral clocks appear to be entrained by food in mammals (Albrecht, 2012) and in fish (López-Olmeda et al., 2010; Feliciano et al., 2011). For instance, food intake has been proven to be a potent synchronizer not only for the liver (Damiola et al., 2000; Stokkan et al., 2001; Kornmann et al., 2007), but also for the heart (Schibler et al., 2003; Mukherji et al., 2015) in mammals. In fish, meal time synchronizes the expression of clock genes in posterior intestine and liver of goldfish (Feliciano et al., 2011; Nisembaum et al., 2012; Tinoco et al., 2014), as well as in heart and fin of zebrafish (Cavallari et al., 2011). These evidences suggest that the feeding schedule has an essential role on the organization of the circadian system in vertebrates, beyond exclusively regulating digestive functions. Although it clearly seems that the interrenal tissue of midday-fed fish is a functional circadian clock, the fact that *clock1a* is not in antiphase with *per1* genes, and *bmal1a* lost its rhythmicity in goldfish fed at mid-scotophase, calls into question the functionality of the clock under this time-lag condition, and support that temporal homeostasis in SF18 animals is altered. Then, the time-lag in feeding schedule may be a stressor for goldfish.

The better adaptation of SF6 fish compared to SF18 is also supported by cortisol results. Our results demonstrate the existence of a daily cortisol rhythm in fish fed at midday, with a peak 5 h before the light onset, which correlates with the functional interrenal clock observed in this group. Conversely, animals fed at the mid-scotophase did not show a daily cortisol rhythm, owing to the fact that the basal levels of this hormone are constantly elevated, being 10 times higher than the basal levels found in midday-fed fish. Such cortisol increase in SF18 fish could be a response to a stressful situation, such as the conflict between environmental cues (light/dark cycle and meal time), that mismatches the phase of hypothalamic, hepatic, and interrenal oscillators. This alteration of circulating cortisol might be due to an altered functionality of the interrenal clock in fish fed at mid-scotophase, in agreement with the hypothesis (under debate) that a local functional clock in the interrenal tissue is necessary to maintain cortisol daily rhythms. In this sense, it is suggested that the adrenal clock could influence the circadian changes in circulating glucocorticoids in mammals (Oster et al., 2006). In fact, fish, and mammals are able to maintain daily cortisol rhythms after an hypophysectomy and in absence of cyclic ACTH levels (Srivastava and Meier, 1972; Meier, 1976), and adrenal clock genes maintain their cyclic expression in rats without a functional hypophysis (Fahrenkrug et al., 2008).

In summary, a time-lag in feeding schedule mismatches clock genes expression in the hypothalamus, the liver, and the interrenal tissue. The increment in cortisol values and the loss of its daily rhythmicity in goldfish fed at mid-scotophase could indicate that these fish are under a stressor. Thus, our results show that the loss of temporal homeostasis can negatively affect the physiology in goldfish and the underlying links between clocks and functional outputs deserve to be explored.

## Author Contributions

MG-B, NdP, and EI conceived and designed the experiments. MG-B, NS, and EI analyzed the samples. All authors participated in sampling animals, interpreted findings, drafted, and revised the manuscript.

## Conflict of Interest Statement

The authors declare that the research was conducted in the absence of any commercial or financial relationships that could be construed as a potential conflict of interest.

## References

[B1] Albrecht U. (2012). Timing to perfection: the biology of central and peripheral circadian clocks. *Neuron* 74 246–260. 10.1016/j.neuron.2012.04.006 22542179

[B2] Aranda A. Madrid J. A. Sánchez-Vázquez F. J. (2001). Influence of light on feeding anticipatory activity in goldfish. *J. Biol. Rhythms* 16 50–57. 10.1177/074873040101600106 11220779

[B3] Azpeleta C. Martínez-Álvarez R. M. Delgado M. J. Isorna E. de Pedro N. (2010). Melatonin reduces locomotor activity and circulating cortisol in goldfish. *Horm. Behav.* 57 323–329. 10.1016/j.yhbeh.2010.01.001 20079741

[B4] Ben-Moshe Z. Alon S. Mracek P. Faigenbloom L. Tovin A. Vatine G. D. (2014). The light-induced transcriptome of the zebrafish pineal gland reveals complex regulation of the circadian clockwork by light. *Nucleic Acids Res.* 42 3750–3767. 10.1093/nar/gkt1359 24423866 PMC3973289

[B5] Cavallari N. Frigato E. Vallone D. Fröhlich N. Lopez-Olmeda J. F. Foà A. (2011). A blind circadian clock in cavefish reveals that opsins mediate peripheral clock photoreception. *PLoS Biol.* 9:e1001142. 10.1371/journal.pbio.1001142 21909239 PMC3167789

[B6] Ceinos R. M. Frigato E. Pagano C. Fröhlich N. Negrini P. Cavallari N. (2018). Mutations in blind cavefish target the light-regulated circadian clock gene, period 2. *Sci. Rep.* 8:8754. 10.1038/s41598-018-27080-2 29884790 PMC5993827

[B7] Challet E. (2015). Keeping circadian time with hormones. *Diabetes Obes. Metab.* 17 76–83. 10.1111/dom.12516 26332971

[B8] Costa L. S. Serrano I. Sánchez-Vázquez F. J. López-Olmeda J. F. (2016). Circadian rhythms of clock gene expression in Nile tilapia (*Oreochromis niloticus*) central and peripheral tissues: influence of different lighting and feeding conditions. *J. Comp. Physiol. B* 186 775–785. 10.1007/s00360-016-0989-x 27085855

[B9] Cowan M. Azpeleta C. López-Olmeda J. F. (2017). Rhythms in the endocrine system of fish: a review. *J. Comp. Physiol. B* 187 1057–1089. 10.1007/s00360-017-1094-5 28447151

[B10] Damiola F. Le Minh N. Preitner N. Kornmann B. Fleury-Olela F. Schibler U. (2000). Restricted feeding uncouples circadian oscillators in peripheral tissues from the central pacemaker in the suprachiasmatic nucleus. *Genes Dev.* 14 2950–2961. 10.1101/gad.183500 11114885 PMC317100

[B11] Delgado M. J. Alonso-Gómez A. L. Gancedo B. de Pedro N. Valenciano A. I. Alonso-Bedate M. (1993). Serotonin N-Acetyltransferase (NAT) activity and Melatonin levels in the frog retina are not correlated during the seasonal cycle. *Gen. Comp. Endocrinol.* 92 143–150. 10.1006/gcen.1993.1151 8282167

[B12] Dibner C. Schibler U. Albrecht U. (2010). The mammalian circadian timing system: organization and coordination of central and peripheral clocks. *Annu. Rev. Physiol.* 72 517–549. 10.1146/annurev-physiol-021909-135821 20148687

[B13] Duggleby R. G. (1981). A nonlinear regression program for small computers. *Anal. Biochem.* 110 9–18. 10.1016/0003-2697(81)90104-4 7212273

[B14] Fahrenkrug J. Hannibal J. Georg B. (2008). Diurnal rhythmicity of the canonical clock genes Per1, Per2 and Bmal1 in the rat adrenal gland is unaltered after hypophysectomy. *J. Neuroendocrinol.* 20 323–329. 10.1111/j.1365-2826.2008.01651.x 18208549

[B15] Feliciano A. Vivas Y. de Pedro N. Delgado M. J. Velarde E. Isorna E. (2011). Feeding time synchronizes clock gene rhythmic expression in brain and liver of goldfish (Carassius auratus). *J. Biol. Rhythms* 26 24–33. 10.1177/0748730410388600 21252363

[B16] Ferrell J. M. Chiang J. Y. L. (2015). Circadian rhythms in liver metabolism and disease. *Acta Pharm. Sin. B* 5 113–122. 10.1016/j.apsb.2015.01.003 26579436 PMC4629216

[B17] Gekakis N. Staknis D. Nguyen H. B. Davis F. C. Wilsbacher L. D. King D. P. (1998). Role of the CLOCK protein in the mammalian circadian mechanism. *Science* 280 1564–1569. 10.1126/science.280.5369.1564 9616112

[B18] Hara R. Wan K. Wakamatsu H. Aida R. Moriya T. Akiyama M. (2001). Restricted feeding entrains liver clock without participation of the suprachiasmatic nucleus. *Genes Cells* 6 269–278. 10.1046/j.1365-2443.2001.00419.x 11260270

[B19] Hastings M. O’Neill J. S. Maywood E. S. (2007). Circadian clocks: regulators of endocrine and metabolic rhythms. *J. Endocrinol.* 195 187–198. 10.1677/JOE-07-0378 17951531

[B20] Herrero M. J. Lepesant J. M. J. (2014). Daily and seasonal expression of clock genes in the pituitary of the European sea bass (*Dicentrarchus labrax*). *Gen. Comp. Endocrinol.* 208 30–38. 10.1016/j.ygcen.2014.08.002 25148807

[B21] Isorna E. de Pedro N. Valenciano A. I. Alonso-Gómez ÁL. Delgado M. J. (2017). Interplay between the endocrine and circadian systems in fishes. *J. Endocrinol* 232 R141–R159. 10.1530/JOE-16-0330 27999088

[B22] Kornmann B. Schaad O. Reinke H. Saini C. Schibler U. (2007). Regulation of circadian gene expression in liver by systemic signals and hepatocyte oscillators. *Cold Spring Harb. Symp. Quant. Biol.* 72 319–330. 10.1101/sqb.2007.72.041 18419289

[B23] Kwon I. Choe H. K. Son G. H. Kim K. (2011). Mammalian molecular clocks. *Exp. Neurobiol.* 20 18–28. 10.5607/en.2011.20.1.18 22110358 PMC3213736

[B24] Lamia K. A. Storch K.-F. Weitz C. J. (2008). Physiological significance of a peripheral tissue circadian clock. *Proc. Natl. Acad. Sci. U.S.A.* 105 15172–15177. 10.1073/pnas.0806717105 18779586 PMC2532700

[B25] Livak K. J. Schmittgen T. D. (2001). Analysis of relative gene expression data using real-time quantitative PCR and the 2^-Δ^ ^Δ^ *^C^*_T_ Method. *Methods* 25 402–408. 10.1006/meth.2001.1262 11846609

[B26] López-Olmeda J. F. Montoya A. Oliveira C. Sánchez-Vázquez F. J. (2009). Synchronization to light and restricted-feeding schedules of behavioral and humoral daily rhythms in gilthead sea bream (*Sparus aurata*). *Chronobiol. Int.* 26 1389–1408. 10.3109/07420520903421922 19916838

[B27] López-Olmeda J. F. Tartaglione E. V. Iglesia H. O. de la Sánchez-Vázquez F. J. (2010). Feeding entrainment of food-anticipatory activity and per1 expression in the brain and liver of zebrafish under different lighting and feeding conditions. *Chronobiol. Int.* 27 1380–1400. 10.3109/07420528.2010.501926 20795882

[B28] Martín-Robles ÁJ. Whitmore D. Sánchez-Vázquez F. J. Pendón C. Muñoz-Cueto J. A. (2012). Cloning, tissue expression pattern and daily rhythms of Period1, Period2, and Clock transcripts in the flatfish Senegalese sole, *Solea senegalensis*. *J. Comp. Physiol. B* 182 673–685. 10.1007/s00360-012-0653-z 22373774

[B29] Meier A. H. (1976). Daily variation in concentration of plasma corticosteroid in hypophysectomized rats. *Endocrinology* 98 1475–1479. 10.1210/endo-98-6-1475 179796

[B30] Mendoza J. Challet E. (2009). Brain clocks: from the suprachiasmatic nuclei to a cerebral network. *Neurosci. Rev. J. Bringing Neurobiol. Neurol. Psychiatry* 15 477–488. 10.1177/1073858408327808 19224887

[B31] Montoya A. López-Olmeda J. F. Garayzar A. B. S. Sánchez-Vázquez F. J. (2010). Synchronization of daily rhythms of locomotor activity and plasma glucose, cortisol and thyroid hormones to feeding in *Gilthead seabream* (*Sparus aurata*) under a light–dark cycle. *Physiol. Behav.* 101 101–107. 10.1016/j.physbeh.2010.04.019 20434474

[B32] Moore H. A. Whitmore D. (2014). Circadian rhythmicity and light sensitivity of the zebrafish brain. *PLoS One* 9:e86176. 10.1371/journal.pone.0086176 24465943 PMC3899219

[B33] Mukherji A. Kobiita A. Chambon P. (2015). Shifting the feeding of mice to the rest phase creates metabolic alterations, which, on their own, shift the peripheral circadian clocks by 12 hours. *Proc. Natl. Acad. Sci. U.S.A.* 112 E6683–E6690. 10.1073/pnas.1519735112 26627259 PMC4672831

[B34] Nader N. Chrousos G. P. Kino T. (2010). Interactions of the circadian CLOCK system and the HPA axis. *Trends Endocrinol. Metab. TEM* 21 277–286. 10.1016/j.tem.2009.12.011 20106676 PMC2862789

[B35] Nakamura K. Inoue I. Takahashi S. Komoda T. Katayama S. (2008). Cryptochrome and Period proteins are regulated by the CLOCK/BMAL1 gene: crosstalk between the PPARs/RXRα-regulated and CLOCK/BMAL1-regulated systems. *PPAR Res.* 2008:348610. 10.1155/2008/348610 18317514 PMC2248703

[B36] Nisembaum L. G. Velarde E. Tinoco A. B. Azpeleta C. de Pedro N. Alonso-Gómez A. L. (2012). Light-dark cycle and feeding time differentially entrains the gut molecular clock of the goldfish (*Carassius auratus*). *Chronobiol. Int.* 29 665–673. 10.3109/07420528.2012.686947 22734567

[B37] Oster H. Damerow S. Kiessling S. Jakubcakova V. Abraham D. Tian J. (2006). The circadian rhythm of glucocorticoids is regulated by a gating mechanism residing in the adrenal cortical clock. *Cell Metab.* 4 163–173. 10.1016/j.cmet.2006.07.002 16890544

[B38] Paredes J. F. López-Olmeda J. F. Martínez F. J. Sánchez-Vázquez F. J. (2015). Daily rhythms of lipid metabolic gene expression in zebra fish liver: response to light/dark and feeding cycles. *Chronobiol. Int.* 32 1438–1448. 10.3109/07420528.2015.1104327 26595085

[B39] Paredes J. F. Vera L. M. Martinez-Lopez F. J. Navarro I. Vázquez F. J. S. (2014). Circadian rhythms of gene expression of lipid metabolism in Gilthead sea bream liver: synchronisation to light and feeding time. *Chronobiol. Int.* 31 613–626. 10.3109/07420528.2014.881837 24517141

[B40] Patiño M. A. L. Rodríguez-Illamola A. Conde-Sieira M. Soengas J. L. Míguez J. M. (2011). Daily rhythmic expression patterns of clock1a, bmal1, and per1 genes in retina and hypothalamus of the rainbow trout, *Oncorhynchus mykiss*. *Chronobiol. Int.* 28 381–389. 10.3109/07420528.2011.566398 21721853

[B41] Ramirez-Plascencia O. D. Saderi N. Escobar C. Salgado-Delgado R. C. (2017). Feeding during the rest phase promotes circadian conflict in nuclei that control energy homeostasis and sleep–wake cycle in rats. *Eur. J. Neurosci.* 45 1325–1332. 10.1111/ejn.13563 28370506

[B42] Refinetti R. (2015). Comparison of light, food, and temperature as environmental synchronizers of the circadian rhythm of activity in mice. *J. Physiol. Sci.* 65 359–366. 10.1007/s12576-015-0374-7 25800223 PMC10717296

[B43] Reppert S. M. Weaver D. R. (2002). Coordination of circadian timing in mammals. *Nature* 418 935–941. 10.1038/nature00965 12198538

[B44] Sánchez J. A. Madrid J. A. Sánchez-Vázquez F. J. (2010). Molecular cloning, tissue distribution, and daily rhythms of expression of per1 gene in European sea bass (*Dicentrarchus labrax*). *Chronobiol. Int.* 27 19–33. 10.3109/07420520903398633 20205555

[B45] Sanchez J. A. Sanchez-Vazquez F. J. (2009). Feeding entrainment of daily rhythms of locomotor activity and clock gene expression in zebrafish brain. *Chronobiol. Int.* 26 1120–1135. 10.3109/07420520903232092 19731109

[B46] Sánchez-Bretaño A. Alonso-Gómez ÁL. Delgado M. J. Isorna E. (2015a). The liver of goldfish as a component of the circadian system: integrating a network of signals. *Gen. Comp. Endocrinol.* 221 213–216. 10.1016/j.ygcen.2015.05.001 25963042

[B47] Sánchez-Bretaño A. Gueguen M.-M. Cano-Nicolau J. Kah O. Alonso-Gómez ÁL. Delgado M. J. (2015b). Anatomical distribution and daily profile of gper1b gene expression in brain and peripheral structures of goldfish (*Carassius auratus*). *Chronobiol. Int.* 32 889–902. 10.3109/07420528.2015.1049615 26171989

[B48] Schibler U. Gotic I. Saini C. Gos P. Curie T. Emmenegger Y. (2015). Clock-talk: interactions between central and peripheral circadian oscillators in mammals. *Cold Spring Harb. Symp. Quant. Biol.* 80 223–232. 10.1101/sqb.2015.80.027490 26683231

[B49] Schibler U. Ripperger J. Brown S. A. (2003). Peripheral circadian oscillators in mammals: time and food. *J. Biol. Rhythms* 18 250–260. 10.1177/0748730403018003007 12828282

[B50] Schmutz I. Albrecht U. Ripperger J. A. (2012). The role of clock genes and rhythmicity in the liver. *Mol. Cell. Endocrinol.* 349 38–44. 10.1016/j.mce.2011.05.007 21664421

[B51] Schreck C. B. Tort L. (2016). “Chapter 1 - The concept of stress in fish,” in *Fish Physiology Biology of Stress in Fish*, eds Schreck C. B. Tort L. Farrell A. P. Brauner C. J. (Cambridge, MA: Academic Press), 1–34. 10.1016/B978-0-12-802728-8.00001-1

[B52] Son G. H. Chung S. Choe H. K. Kim H.-D. Baik S.-M. Lee H. (2008). Adrenal peripheral clock controls the autonomous circadian rhythm of glucocorticoid by causing rhythmic steroid production. *Proc. Natl. Acad. Sci. U.S.A.* 105 20970–20975. 10.1073/pnas.0806962106 19091946 PMC2634940

[B53] Spencer R. L. Chun L. E. Hartsock M. J. Woodruff E. R. (2018). Glucocorticoid hormones are both a major circadian signal and major stress signal: how this shared signal contributes to a dynamic relationship between the circadian and stress systems. *Front. Neuroendocrinol.* 49:52–71. 10.1016/j.yfrne.2017.12.005 29288075

[B54] Srivastava A. K. Meier A. H. (1972). Daily variation in concentration of cortisol in plasma in intact and hypophysectomized gulf killifish. *Science* 177 185–187. 10.1126/science.177.4044.185 4339354

[B55] Stephan F. K. (2002). The “Other” circadian system: food as a zeitgeber. *J. Biol. Rhythms* 17 284–292. 10.1177/074873040201700402 12164245

[B56] Stokkan K.-A. Yamazaki S. Tei H. Sakaki Y. Menaker M. (2001). Entrainment of the circadian clock in the liver by feeding. *Science* 291 490–493. 10.1126/science.291.5503.490 11161204

[B57] Tinoco A. B. Nisembaum L. G. de Pedro N. Delgado M. J. Isorna E. (2014). Leptin expression is rhythmic in brain and liver of goldfish (*Carassius auratus*). *Role Feed. Time. Gen. Comp. Endocrinol.* 204 239–247. 10.1016/j.ygcen.2014.06.006 24932715

[B58] Tsang A. H. Barclay J. L. Oster H. (2014). Interactions between endocrine and circadian systems. *J. Mol. Endocrinol.* 52 R1–R16. 10.1530/JME-13-0118 23997239

[B59] Vatine G. Vallone D. Gothilf Y. Foulkes N. S. (2011). It’s time to swim! Zebrafish and the circadian clock. *FEBS Lett.* 585 1485–1494. 10.1016/j.febslet.2011.04.007 21486566

[B60] Velarde E. Haque R. Iuvone P. M. Azpeleta C. Alonso-Gómez A. L. Delgado M. J. (2009). Circadian clock genes of goldfish, *Carassius auratus*: cDNA cloning and rhythmic expression of period and Cryptochrome transcripts in retina, liver, and gut. *J. Biol. Rhythms* 24 104–113. 10.1177/0748730408329901 19346448 PMC2666933

[B61] Vera L. M. de Pedro N. Gómez-Milán E. Delgado M. J. Sánchez-Muros M. J. Madrid J. A. (2007). Feeding entrainment of locomotor activity rhythms, digestive enzymes and neuroendocrine factors in goldfish. *Physiol. Behav.* 90 518–524. 10.1016/j.physbeh.2006.10.017 17196229

[B62] Vera L. M. Negrini P. Zagatti C. Frigato E. Sánchez-Vázquez F. J. Bertolucci C. (2013). Light and feeding entrainment of the molecular circadian clock in a marine teleost (*Sparus aurata*). *Chronobiol. Int.* 30 649–661. 10.3109/07420528.2013.775143 23688119

[B63] Vivas Y. Azpeleta C. Feliciano A. Velarde E. Isorna E. Delgado M. J. (2011). Time-dependent effects of leptin on food intake and locomotor activity in goldfish. *Peptides* 32 989–995. 10.1016/j.peptides.2011.01.028 21291931

[B64] Welsh D. K. Takahashi J. S. Kay S. A. (2010). Suprachiasmatic nucleus: cell autonomy and network properties. *Annu. Rev. Physiol.* 72 551–577. 10.1146/annurev-physiol-021909-135919 20148688 PMC3758475

